# Surface Patterning of Closed Nanochannel Using VUV Light and Surface Evaluation by Streaming Current

**DOI:** 10.3390/mi12111367

**Published:** 2021-11-06

**Authors:** Kyojiro Morikawa, Haruki Kazumi, Yoshiyuki Tsuyama, Ryoichi Ohta, Takehiko Kitamori

**Affiliations:** 1 Department of Applied Chemistry, School of Engineering, The University of Tokyo, 7-3-1 Hongo, Bunkyo, Tokyo 113-8656, Japan; kazumi-haruki@g.ecc.u-tokyo.ac.jp (H.K.); ohta@icl.t.u-tokyo.ac.jp (R.O.); 2 Department of Bioengineering, School of Engineering, The University of Tokyo, 7-3-1 Hongo, Bunkyo, Tokyo 113-8656, Japan; ytsuyama@g.ecc.u-tokyo.ac.jp; 3 Collaborative Research Organization for Micro and Nano Multifunctional Devices (NMfD), The University of Tokyo, 7-3-1 Hongo, Bunkyo, Tokyo 113-8656, Japan; 4 Institute of Nanoengineering and Microsystems (iNEMS), Department of Power Mechanical Engineering, National Tsing Hua University, No. 101, Section 2, Kuang-Fu Road, Hsinchu 300044, Taiwan

**Keywords:** nanofluidics, nanofabrication, surface patterning, streaming current, Laplace valve

## Abstract

In nanofluidics, surface control is a critical technology because nanospaces are surface-governed spaces as a consequence of their extremely high surface-to-volume ratio. Various surface patterning methods have been developed, including patterning on an open substrate and patterning using a liquid modifier in microchannels. However, the surface patterning of a closed nanochannel is difficult. In addition, the surface evaluation of closed nanochannels is difficult because of a lack of appropriate experimental tools. In this study, we verified the surface patterning of a closed nanochannel by vacuum ultraviolet (VUV) light and evaluated the surface using streaming-current measurements. First, the C_18_ modification of closed nanochannels was confirmed by Laplace pressure measurements. In addition, no streaming-current signal was detected for the C_18_-modified surface, confirming the successful modification of the nanochannel surface with C_18_ groups. The C_18_ groups were subsequently decomposed by VUV light, and the nanochannel surface became hydrophilic because of the presence of silanol groups. In streaming-current measurements, the current signals increased in amplitude with increasing VUV light irradiation time, indicating the decomposition of the C_18_ groups on the closed nanochannel surfaces. Finally, hydrophilic/hydrophobic patterning by VUV light was performed in a nanochannel. Capillary filling experiments confirmed the presence of a hydrophilic/hydrophobic interface. Therefore, VUV patterning in a closed nanochannel was demonstrated, and the surface of a closed nanochannel was successfully evaluated using streaming-current measurements.

## 1. Introduction

Recent progress in the miniaturization of fluidic devices has led to the downsizing of microfluidics to nanofluidics. A nanochannel has a typical diameter of 10–1000 nm and a volume on the femtoliter to picoliter scale. Such ultrasmall channels have led to the development of various new functional devices [1,2,3,4,5]. The basic technologies used to develop these functional devices include fabrication, flow control, and detection. During fabrication, in particular, surface modification is critical. The surface-to-volume (S/V) ratio in a typical nanospace is extremely high, which means that molecules/ions on nanochannel surfaces strongly affect the behavior of the liquid phase. For example, dissociated protons from silanol groups on nanochannel surfaces were found to dramatically change the concentration of liquids in the nanochannels [6,7], resulting in a pH value of ~5 when a neutral solution was introduced into the nanochannels [8,9]. The high S/V ratio also contributes to liquid motion in nanochannels. In accordance with the Young–Laplace equation, liquids in nanochannels can be moved by surface tension at the solid/liquid interface, which depends on the nanochannel size. In a hydrophilic nanochannel, the capillary pressure was found to reach the sub-megapascal order, which was confirmed by capillary filling experiments in nanochannels [10,11]. In the case of hydrophobic surfaces, the acceleration and deceleration of liquids—even a stop-and-go motion of the liquids—can be induced in nanochannels [12,13]. The surface effect is enhanced by the nanostructure of the nanochannel surfaces, which has led to more sophisticated fluidic control [14,15]. In addition, molecules/ions on the nanochannel surfaces strongly affect molecules in the liquid phase. Given that the diffusion length for typical molecules such as proteins in liquids is in the order of 10 μm within 1 s, molecules in the liquid phase of the nanochannel can frequently contact nanochannel surfaces. By exploiting such characteristics in nanochannels, researchers have developed various novel devices with functionalized nanochannel surfaces. For example, researchers have developed enzymatic reaction devices [16,17], immunochemical reaction devices [18,19], and bioelectrochemical reaction devices [20] by effectively utilizing reactions at the solid/liquid interfaces in nanochannels. In addition, our group recently found that even small amounts of ions that remained on nanochannel surfaces after the channel fabrication processes reacted with molecules in the liquid phase, which affected the detection of signals from the main chemical reaction [21]. Thus, nanospaces are surface-governed spaces, and surface control is therefore essential for nanofluidics.

Channel surface modification and patterning methods have been developed to functionalize surfaces. Among patterning methods for open substrates, nanoimprint lithography [22,23,24] and combined processes of lithography and lift-off [25,26,27,28] are well known. In addition, some groups have developed devices by bonding a patterned substrate and another substrate fabricated in a channel [12,29,30,31]. For nanochannel surface patterning, a method that combines vacuum ultraviolet (VUV) light patterning [18] and low-temperature bonding [32] was developed. However, with such patterning methods, only the top surface and/or bottom surface can be modified by functional molecules. This limitation is problematic for patterning the entire surface (i.e., top, bottom, and side surfaces) of nanochannels. For example, the hydrophobic modification of the entire surface is required for a hydrophobic Laplace valve, because liquid leakage occurs from hydrophilic parts if the hydrophilic part remains on some surfaces. One method for modifying the entire surface is to introduce modifier liquids into the closed channels [33]. For example, partial modification was used to induce multiphase flow of modifier liquids [34,35]. Using this method, Nakao et al. developed a novel nanofluidic device for a single-cell analysis [36] patterned by several functional surfaces. Modification methods are not limited to the introduction of liquids. Partial polymerization was achieved by introducing O_2_ gas to partially inhibit a polymerization reaction [37]. Surface modification has also been achieved by plasma treatment of a channel by introducing O_2_ plasma into it [38,39]. Even with these methods, complicated patterns such as stripes across the flow direction are difficult to form because the flow is basically continuous. Therefore, the surface patterning of closed channels is still difficult, and an effective method for patterning closed channels is needed.

One approach to address surface patterning is photopatterning [40]. In photopatterning, a device fabricated from transparent materials such as glass is used to introduce light into the channel; as a result, the channel surface is activated by photochemical reactions. In this method, the pattern can be easily designed via photomasking. For example, polymerization by UV light was carried out in part of a microchannel by partitioning with a photomask [41,42,43]. These technologies are highly useful for use as surface patterning methods for microchannel surfaces. However, photo-based patterning for nanochannel surfaces has not been reported. Because of the small dimensions of nanochannels, photochemical reactions within them are controlled by still-unknown factors.

Surface evaluations to check whether small and closed nanochannels have been successfully patterned are also difficult. For bulk samples, numerous tools are used to evaluate the surface, including contact-angle measurements and X-ray photoelectron spectroscopy (XPS); however, such methods are difficult to apply to closed channels. One approach to evaluating closed channels is to attach fluorescent molecules to their surface [18,44]. However, in these methods, fluorescent probes should be introduced in the channel. Thus, another approach has been proposed: a streaming current. A streaming current is generated by pressure-driven flow in small spaces, and the magnitude of the current depends on the surface charges [45,46]. In this method, the streaming-current signals can be obtained just by water flow, which indicates that the addition of any probe is not necessary and contamination of the channel by the probe does not occur. Previously, we developed methods for measuring the streaming current in a nanochannel [47] and found that the current signal properly reflected the surface charges [48]. Thus, the difference in surface charges before/after surface modification can be evaluated using streaming currents.

In the present study, we examined VUV-light-induced surface patterning of a nanochannel. First, surfaces of a closed nanochannel fabricated on fused-silica substrates were hydrophobically modified with C_18_ groups. The C_18_ groups were then decomposed by VUV light, and the surface was rendered hydrophilic through functionalization with silanol groups. During irradiation with VUV light, reactive oxygen species generated via O_2_ gas molecules absorbing high-energy VUV light oxidatively decomposed the C_18_ molecules on the nanochannel surfaces [34,49]. In this experiment, the dependence on the VUV light irradiation time was varied. Principally, a C_18_ group has no charge, whereas a silanol group has a negative charge because of proton disassociation when exposed to a liquid. We used streaming-current measurements to evaluate the difference in the surface charge before and after the modification. Finally, we demonstrated hydrophobic/hydrophilic patterning in a closed nanochannel by the patterning of C_18_/silanol groups and verified the patterning via capillary-filling experiments.

## 2. Materials and Methods

### 2.1. Hydrophobic/Hydrophilic Patterning Using VUV Light

**Figure 1:**
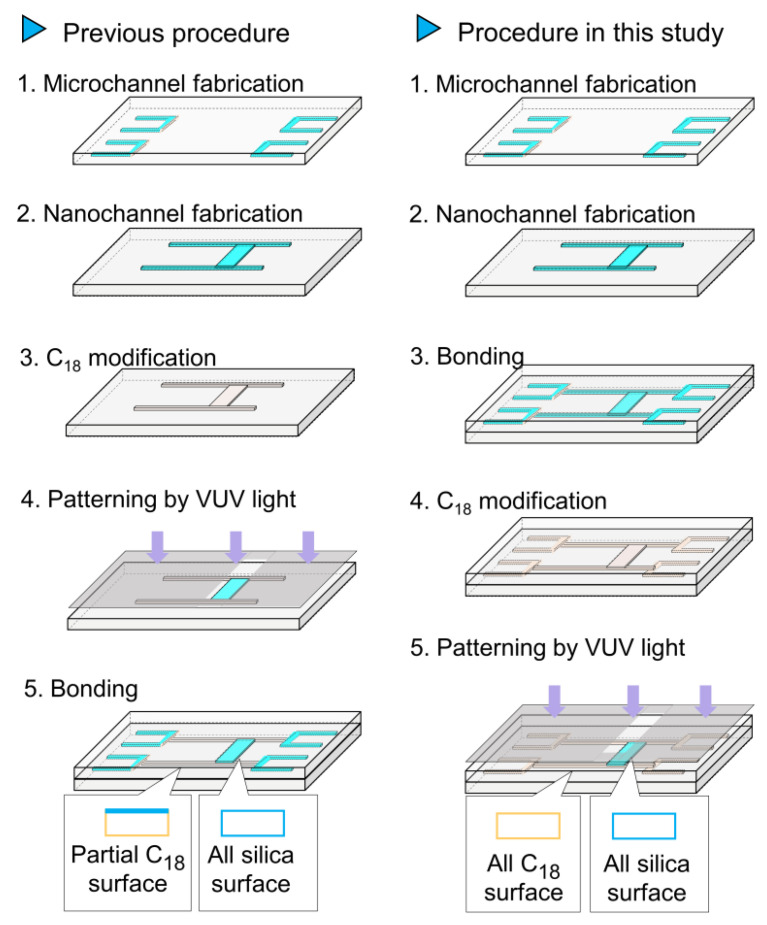
Procedure for nanochannel fabrication and surface patterning.

Figure 1 shows the hydrophobic/hydrophilic patterning procedure. In our previous method, C_18_ modification and VUV light patterning were performed before bonding. As a result, some channels had a partial C_18_ surface (only the bottom and side surfaces were modified with C_18_ groups, as shown in Figure 1, left). In the present study, after the channel fabrication and bonding procedures, the specimens were subjected to C_18_ modification and VUV light patterning. In this procedure, nanochannels with all-silica surfaces and nanochannels with all-C_18_ surfaces were patterned. The details are as follows. Nanochannels were fabricated on a fused-silica substrate by electron-beam (EB) lithography and dry etching [50,51]. First, a Cr layer was deposited onto a fused-silica substrate (VIO-SILSX, Shin-Etsu Quartz, Tokyo, Japan; 70 × 30 × 0.7 mm^3^) and an EB resist (ZEP-520A, Zeon, Tokyo, Japan) was spin-coated onto the Cr layer. After EB lithography (ELS-7500, Elionix, Tokyo, Japan), the EB resist was developed with *o*-xylene and the Cr layer was wet-etched using a Cr etchant solution (Ce(NH_4_)_2_(NO_3_)_6_, FUJIFILM Wako Pure Chemical, Osaka, Japan). Dry etching was performed using a dry-etching apparatus (NE-550, ULVAC, Kanagawa, Japan). On another fused-silica substrate, microchannels for introducing liquid into a nanochannel were fabricated. First, 1,1,1,3,3,3-hexamethyldisilazane (HMDS, Wako Pure Chemical Industries, Osaka, Japan) was spin-coated, followed by spin-coating of a photoresist (KMPR^®^ 1035, Microchem, Round Rock, TX, USA). After photolithography, dry etching was performed to form microchannels. After the channels were fabricated, the two substrates were bonded together with their nanochannels and microchannels aligned. After the bonding process, all the nanochannels were modified with C_18_ using octadecyldimethyl-*N*,*N*-diethylaminosilane (ODS-DEA) synthesized from diethylamine and ODS-Cl (Sigma-Aldrich, St. Louis, MO, USA). After the C_18_ modification process, the nanochannel was modified with chlorotrimethylsilane (Tokyo Chemical Industry, Tokyo, Japan) for end-capping. The nanofluidic device was then irradiated using a VUV-light irradiation system with a wavelength of 172 nm (SUS713, Ushio, Tokyo, Japan).

### 2.2. Streaming-Current Measurements

**Figure 2:**
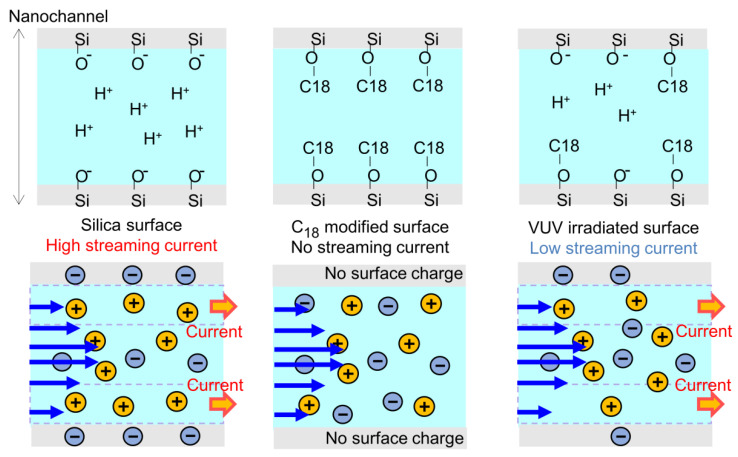
Principle of streaming current corresponding to surface charge.

Figure 2 shows the principle of the streaming current corresponding to the surface charges in a nanochannel. In a fused-silica nanochannel, the silanol groups on a surface dissociate in a liquid phase. The negatively charged surface and the accumulated positive ions near the surface form an electric double layer (EDL). The properties of the liquid in the nanochannel are strongly affected by the EDL because the thickness of the EDL is approximately 1–100 nm. Upon the application of external pressure, the pressure-driven flow of the liquid induces an ion flow in the EDL, which produces a streaming current. After the nanochannel surface is modified with C_18_, it lacks charges because of the conversion of silanol groups to C_18_ groups. In this case, no streaming current is generated. Under irradiation with VUV light, the C_18_ groups are decomposed by the VUV light and the silanol groups are partially regenerated. The streaming current depends on the zeta potential of the surface and its surface charge density [52,53]; therefore, the ratio of recovered silanol groups can be estimated. The details of the streaming-current measurement system are available elsewhere [47,48]. Currents were measured under applied pressures of 0, 0.1, 0.2, and 0.3 MPa. In addition, different surfaces were prepared by varying the VUV light irradiation time from 0 to 17, 51, and 119 min; the streaming currents for each surface were subsequently measured.

## 3. Results and Discussion

**Figure 3:**
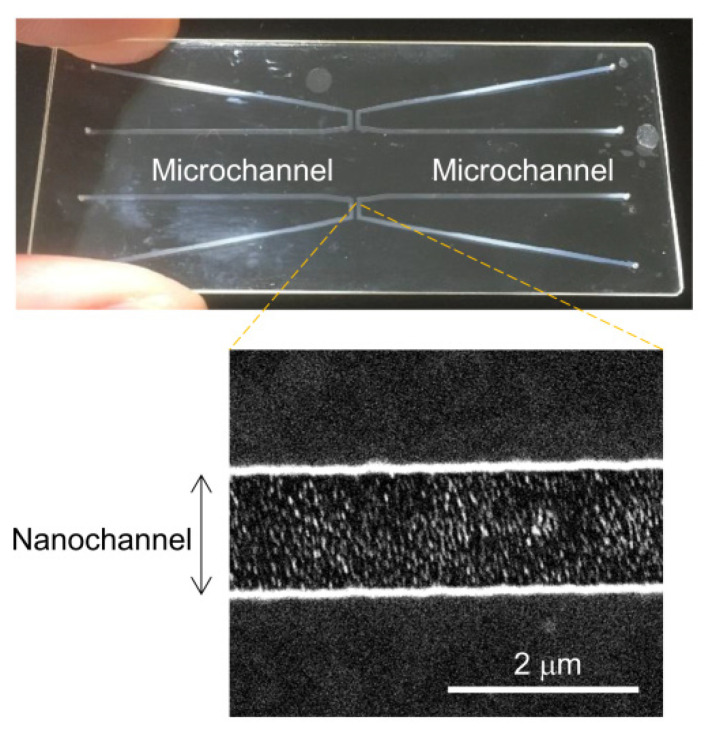
Images of fabricated device and nanochannel for streaming-current measurements.

**Figure 4:**
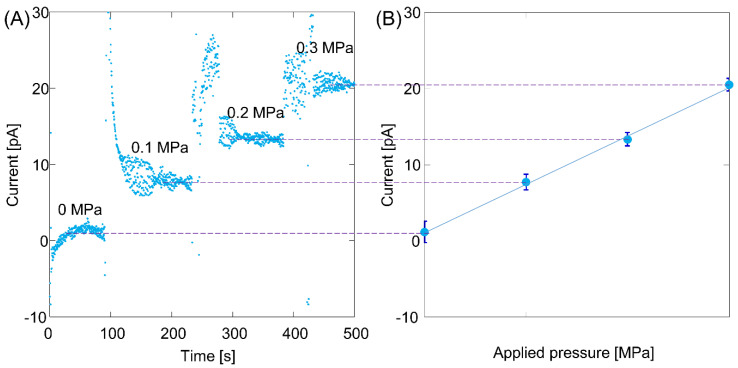
Streaming-current signals for unmodified nanochannel surface: (**A**) raw data for current signals and (**B**) pressure dependence of current.

Figure 3 shows the fabricated nanofluidic device used to verify the C_18_ modification and silanol recovery under irradiation with VUV light. The width and depth of the nanochannel were 1380 and 1050 nm, respectively. The results of streaming-current measurements before the C_18_ modification are shown in Figure 4A. For the first 100 s, current signals were measured in the absence of applied pressure. Thereafter, a pressure of 0.1 MPa was applied. For dozens of seconds after the pressure was applied, the current signals fluctuated, indicating that flow in the nanochannel was not stable. The current signals stabilized thereafter. In a same manner, the applied pressure was increased to 0.2 and 0.3 MPa, and the current signal at each applied pressure was acquired. The obtained current signals (average ± 3σ) were plotted as a function of applied pressure (Figure 4B). A linear relationship was obtained between the applied pressure and the current signals, indicating a successful measurement because they theoretically exhibited a linear relationship. The slope in Figure 4B was recorded as the streaming current normalized by the pressure.

**Figure 5:**
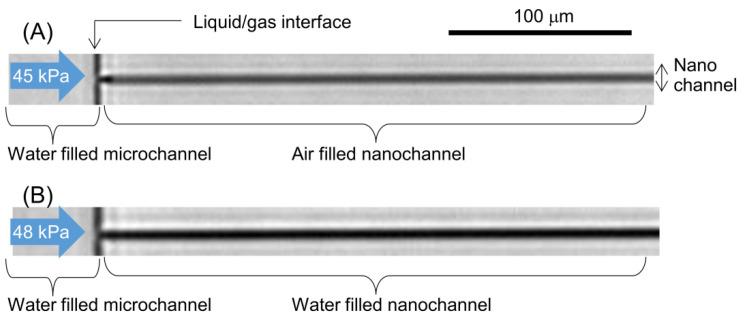
Images of water introduction into hydrophobic nanochannel: (**A**) before water was introduced under an applied pressure of 45 kPa; (**B**) after water was introduced under an applied pressure of 48 kPa.

The nanochannel was next modified with C_18_. To verify the C_18_ modification, we measured the Laplace pressure (hydrophobic pressure) in the nanochannel. Figure 5A shows water introduced under an applied pressure of 45 kPa. The water was stopped at the interface between the microchannel and the nanochannel due to the Laplace pressure. Figure 5B shows water introduction under 48 kPa of applied pressure. Under this condition, the visual appearance of the nanochannel differed from that in Figure 5A because of the difference in refractive index between the air and water. Therefore, 48 kPa was the breaking pressure for the Laplace pressure of the nanochannel. As a result, water was introduced into the nanochannel. Given that the advancing contact angle on a C_18_ surface is 104 ± 4° [15], the calculated Laplace pressure was 58 ± 16 kPa, which indicates that the Laplace pressure obtained from experiments corresponded well with the calculated value. Thus, C_18_ modification of the nanochannel was successful.

**Figure 6:**
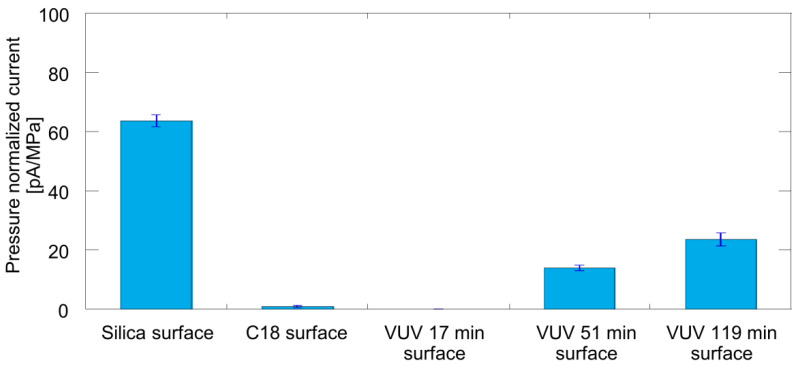
Comparison of streaming current corresponding to different nanochannel surfaces.

Streaming-current measurements were performed using the C_18_-modified nanofluidic device and also using the device after irradiation with VUV light. Figure 6 shows the pressure-normalized streaming-current values for the unmodified and modified nanochannel surfaces. Compared with the current on a fused-silica surface before C_18_ modification, the current on the C_18_-modified surface was greatly decreased to approximately zero. This result also indicates that the C_18_ modification was successful: no streaming current was observed because of a lack of surface charges on the C_18_ groups. For the VUV-irradiated specimens, the current increased with increasing VUV irradiation time. These results are consistent with surface charges being recovered upon C_18_ decomposition under VUV light. After 119 min of irradiation, the current was 37% of that on an unmodified fused-silica surface. These results suggested that apporoximately 37% of the silanol groups were recovered after 119 min of VUV irradiation. However, the estimation of the recovery ratio needs further investigation. Using bulk chemical parameters to calculate the surface charge density is difficult because the properties of liquids in nanochannels differ from their bulk properties. For example, compared with bulk liquids, liquids in nanochannels have been reported to exhibit a lower pH [8,9], lower dielectric constant [9,54], and enhanced proton dissociation [48]. Therefore, obtaining an accurate recovery ratio is difficult. Nonetheless, the phenomenon of surface charges being recovered upon the conversion of C_18_ to silanol groups was confirmed. To increase the recovery ratio, a longer irradiation time, an increase in light power, and the optimization of O_2_ concentration were considered. In addition, the streaming current was successfully measured in 50 nm channels [51] at the minimum; thus, the surface evaluation method in the present study is applicable for smaller nanochannels.

In our previous experiments, we found that almost the entire surface was recovered in approximately 12 min under VUV-light irradiation of the open substrate on an aminopropyltriethoxysilane (APTES)-functionalized surface [18]. To explain the difference between the effect of VUV light on the APTES-functionalized and C_18_-functionalized surfaces, we considered two possibilities. The first is that the difference is attributable to the differences between the properties of molecules on the substrate surface. APTES has a three-carbon chain, whereas C_18_ has an 18-carbon chain; the decomposition of C_18_ would reasonably require a longer irradiation time than the decomposition of APTES. The second possibility is that the difference is attributable to the difference in reaction fields. In a nanochannel, O_2_ molecules are not readily provided because of their small diffusion flux. Consequently, a prolonged time was needed for recovery from the C_18_ functionalization of the nanochannel surfaces compared with the time need for recovery from APTES on open substrate surfaces. The results and discussion in the present study are useful for understanding chemical reactions at the gas/solid interface in a nanochannel.

**Figure 7:**
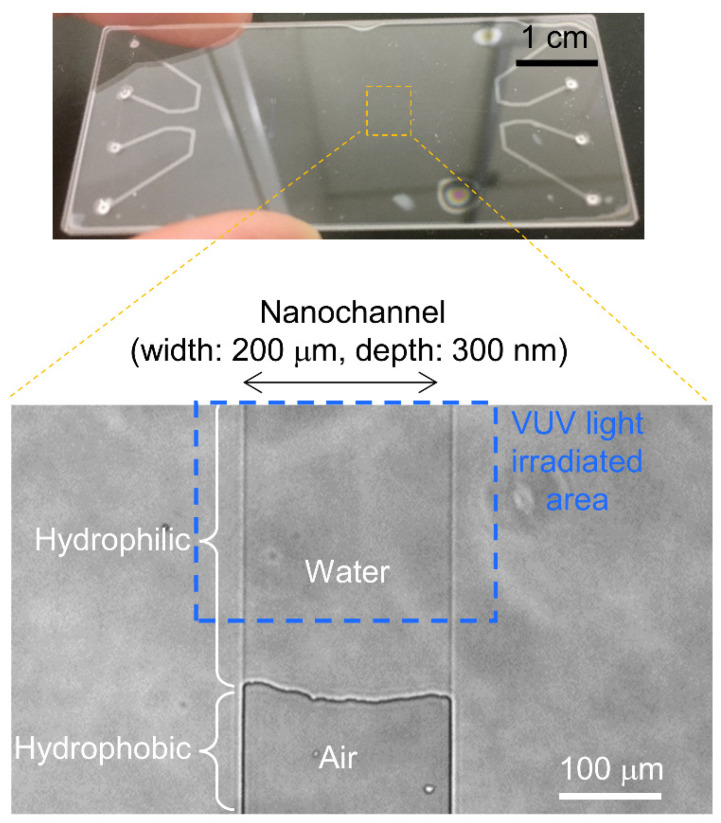
Images of hydrophobic/hydrophilic patterned nanofluidic device and result of water filling experiment.

Finally, liquid introduction was demonstrated using a VUV-light-patterned nanochannel. As shown in Figure 7, the 200 μm wide and 300 nm deep channel was fabricated. After C_18_ modification of all the nanochannels, a part of the 300 nm channel area was irradiated by VUV light for 17 min. After the VUV light irradiation, water was introduced under an applied pressure of 90 kPa; the water only filled the hydrophilic area and stopped at the hydrophilic/hydrophobic interface. There was a gap of approximately 100 μm between the edge of VUV light irradiated area and hydrophilic/hydrophobic interface. It was considered that excited O_2_ gas molecules were diffused during the VUV light irradiation, and their diffusion lengths were to a similar scale as our previous results [18]. From the results, to produce hydrophilic surfaces, it was confirmed that 17 min of VUV irradiation was enough, and more hydrophilic surfaces can be principally considered in the case of a longer VUV irradiation time. In light of the fact that that no streaming-current signals in 17 min of VUV irradiation were obtained, the following suggestion was considered: carbon structures in C_18_ were partially decomposed by VUV light, and the remaining partial carbon structure on the surface showed hydrophilic patterning, but a streaming current was not observed because they did not induce a surface charge. On the basis of this discussion, useful practical information was obtained as follows. To produce hydrophilic/hydrophobic patterning alone, 17 min VUV irradiation is required. In addition, to produce silanol/C_18_ patterning, a longer VUV irradiation time is required, as shown in streaming-current results with 51 min and 119 min VUV irradiation. When the applied pressure was increased to 95 kPa, the gas/liquid interface was moved to the gas area and water filled the hydrophobic area. The breaking pressure of 95 kPa was reasonable compared to the calculated Laplace pressure of 116 ± 33 kPa using a channel width of 200 μm, a depth of 300 nm and a contact angle of 104 ± 4° [15]. Therefore, the proposed VUV patterning method achieved hydrophilic/hydrophobic patterning. This work thus represents the first report of hydrophilic/hydrophobic patterning of a closed nanochannel by VUV light. Principally, the irradiation of VUV light was considered to be independent of channel width; thus, this method is promising for the patterning of a shallower channel such as a square-type nanochannel in which width and depth are at the 100 nm scale.

## 4. Conclusions

In the present study, we verified the surface patterning of a closed nanochannel by VUV light. First, surfaces of a closed nanochannel fabricated on fused-silica substrates were hydrophobically modified with C_18_. The C_18_ modification was confirmed by Laplace pressure measurements, and the results showed a reasonable Laplace pressure compared with the calculated pressure. In addition, no streaming-current signal was obtained for the C_18_ surface, further indicating the successful modification of the surface with C_18_ groups. The C_18_ groups were subsequently decomposed by VUV light, and the surface became hydrophilic in nature because of the formation of silanol groups. Streaming-current measurements showed that the current signals increased in intensity with increasing VUV light irradiation time, indicating the decomposition of the C_18_ groups on closed nanochannel surfaces. Compared with the streaming-current signals before C_18_ modification, 37% of the signal was recovered after 119 min of VUV irradiation. To increase the recovery ratio, a longer irradiation time, an increase in light power, and the optimization of O_2_ concentration were considered. In addition, on the basis of our previous streaming-current results, the surface evaluation method in the present study is applicable for 50 nm channels at the minimum. Further investigations are needed to estimate the recovery ratio of surface charge on the nanochannel on the basis of the unique properties of liquids in a nanochannel compared with those of the corresponding bulk liquids. Hydrophilic/hydrophobic patterning by VUV light was performed in a nanochannel. Capillary-filling experiments confirmed the formation of a hydrophilic/hydrophobic interface. The results showed that 17 min of VUV irradiation was required to produce hydrophilic/hydrophobic patterning, and VUV irradiation longer than 51 min was required to produce silanol/C_18_ patterning. In summary, VUV patterning in a closed nanochannel was demonstrated for the first time, and the surface of a closed nanochannel was successfully evaluated using streaming-current measurements.
